# Assessment of diffuse myocardial fibrosis in systemic sclerosis by cardiac magnetic resonance imaging

**DOI:** 10.1186/1532-429X-15-S1-E121

**Published:** 2013-01-30

**Authors:** Daniel C Lee, Roberto Sarnari, Brandon Benefield, Alejandro Aquino, James Carr, John Varga, Monique Hinchcliff, Edwin Wu, Sanjiv Shah

**Affiliations:** 1 Department of Medicine, Northwestern University, Feinberg School of Medicine, Chicago, IL, USA; 2 Department of Radiology, Northwestern University, Feinberg School of Medicine, Chicago, IL, USA; 3 Feinberg Cardiovascular Research Institute, Northwestern University, Feinberg School of Medicine, Chicago, IL, USA

## Background

Systemic sclerosis (SSc) is a connective tissue disorder characterized by excessive fibrosis of the skin and internal organs, including the heart. Traditional late gadolinium enhancement (LGE) by cardiovascular magnetic resonance (CMR) imaging is dependent on differential (i.e., focal) fibrosis within the myocardium. However, diffuse interstitial myocardial fibrosis (DF) affecting the entire myocardium may be missed because no normal reference region exists. T1 mapping has been utilized in a variety of techniques to assess diffuse fibrosis: 1) pre-contrast T1 measurement (PreT1), 2) post-contrast T1 measurement (PostT1), 3) partition coefficient (PC), and 4) volume of distribution (VD). We sought to evaluate the accuracy of LGE and various T1 mapping techniques for the assessment of myocardial fibrosis in patients with SSc.

## Methods

CMR was performed in 13 SSc patients (5 diffuse and 8 limited cutaneous) and 13 age-matched controls. Cine, pre- and post-contrast T1 mapping, and late gadolinium enhanced (LGE) imaging was performed. Calculation of PC and VD were: PC = ΔR1myocardium/ΔR1bloodpool. VD = [ΔR1myocardium/ΔR1bloodpool × p × (1 - hematocrit)] - Vp, where R1 = 1/ T1, ΔR1 is post-contrast - pre-contrast R1, p is myocardial specific density (1.05), and Vp is myocardial plasma volume fraction (0.045). The modified Rodnan skin score (mRSS), a measure of SSc disease activity, was quantified in all SSc patients by clinicians blinded to all CMR data.

## Results

**Figure 1:**
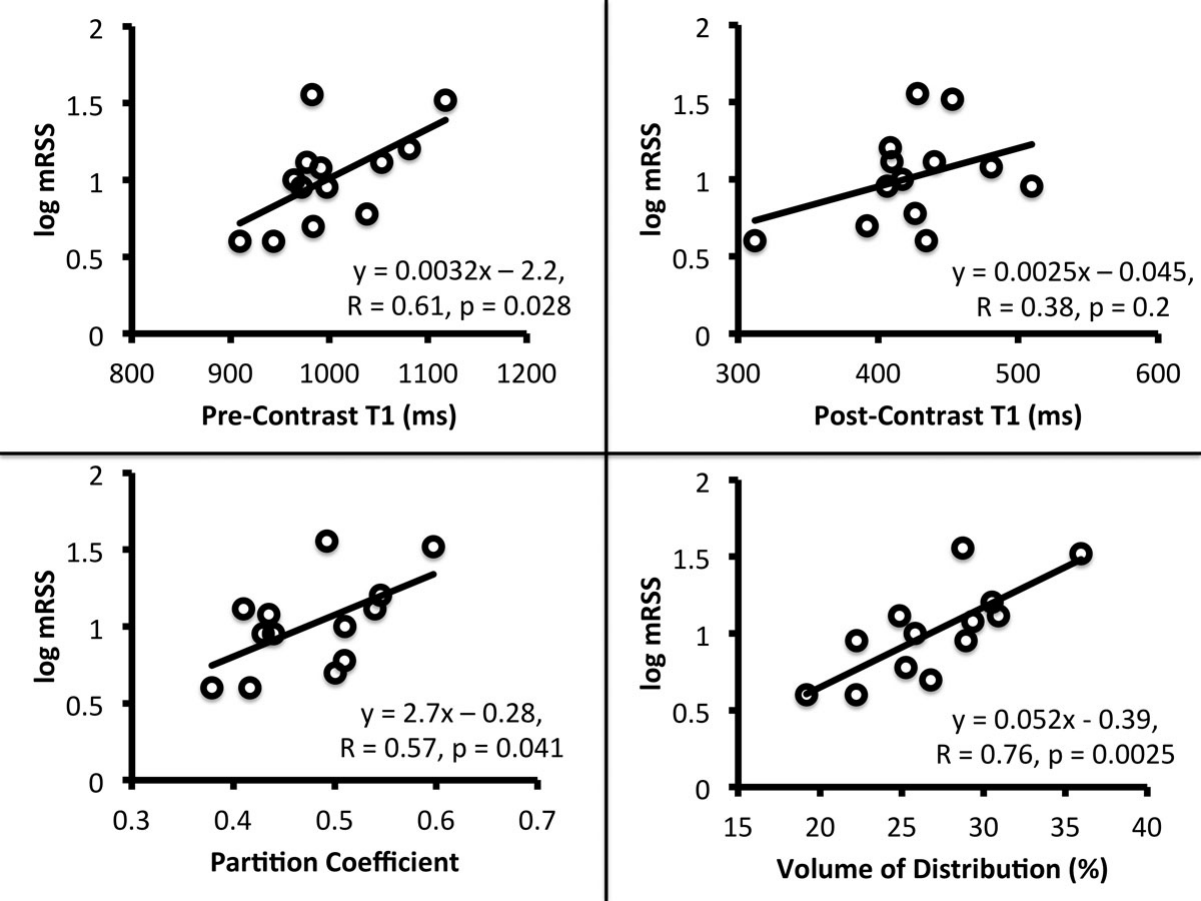
Scatter plots for CMR measures of diffuse fibrosis versus scleroderma severity by mRSS

There was no visible LGE in 10/13 SSc and 13/13 controls. VD was significantly higher in SSc than controls (27.4 ± 4.6% vs. 20.6 ± 3.3%, p = 0.0003), even when patients with visible LGE were excluded (26.9 ±4.0% vs. 20.6 ± 3.3%, p = 0.001). PreT1, PC, and VD all correlated significantly with log mRSS in SSc patients, the correlation for PostT1 was not significant (Figure 1 and Table 1). Comparison of correlation coefficients demonstrates VD > PreT1 > PD > PostT1.

**Table 1:** Correlation with Systemic Sclerosis Severity by mRSS

CMR Parameter	Equation	R value	p-value
Pre-Contrast T1	y = 0.0032x - 2.2	0.61	0.028
Post-Contrast T1	y = 0.0025x - 0.045	0.38	0.20
Partition Coefficient	y = 2.7x - 0.28	0.57	0.041
Volume of Distribution	y = 0.052x - 0.39	0.76	0.0025

## Conclusions

This is the first study to compare various T1 mapping techniques for the assessment of diffuse interstitial myocardial fibrosis in patients with SSc. PreT1 provides a surprisingly good assessment of DF in SSc, and may be useful in patients with impaired renal function. Calculation of VD - which requires measurement of hematocrit, PreT1, and PostT1 - is more time consuming but provides the most accurate assessment of DF in SSc. Calculation of PC and PostT1 - which allow one to eliminate hematocrit or hematocrit and PreT1, respectively - provide simpler but less accurate measures of DF in SSc.

## Funding

None.

